# Exploring households’ resilience to climate change-induced shocks using Climate Resilience Index in Dinki watershed, central highlands of Ethiopia

**DOI:** 10.1371/journal.pone.0219393

**Published:** 2019-07-09

**Authors:** Mengistu Asmamaw, Seid Tiku Mereta, Argaw Ambelu

**Affiliations:** 1 Department of Environmental Health Science and Technology, Jimma University, Jimma, Ethiopia; 2 Department of Biology, Debre Berhan University, Debre Berhan, Ethiopia; University of Florida, UNITED STATES

## Abstract

This study assessed households’ resilience to climate change-induced shocks in Dinki watershed, northcentral highlands of Ethiopia. The data were collected through a cross-sectional survey conducted on 288 households, three focus group discussions, and 15 key informant interviews. The Climate Resilience Index (CRI) based on the three resilience capacities (absorptive, adaptive and transformative) frame was used to measure households’ resilience to climate change-induced shocks on an agro-ecological unit of analysis. A principal component analysis (PCA) and multiple regression analysis were used to identify determinant factors and indicators to households’ resilience, respectively. Findings indicate that the indexed scores of major components clearly differentiated the study communities in terms of their agro-ecological zones. Specifically, the absorptive capacity (0.495) was the leading contributing factor to resilience followed by adaptive (0.449) and transformative (0.387) capacities. Likewise, the Midland was relatively more resilient with a mean index value of 0.461. Both the PCA and multiple regression analysis indicated that access to and use of livelihood resources, such as farmlands and livestock holdings, diversity of income sources, infrastructure and social capital were determinants of households’ resilience. In general, it might be due to their exposure to recurrent shocks coupled with limited adaptive capacities including underdeveloped public services, poor livelihood diversification practices, among others, the study communities showed minimal resilience capacity with a mean score of 0.44. Thus, in addition to short-term buffering strategies, intervention priority focusing on both adaptive and transformative capacities, particularly focusing on most vulnerable localities and constrained livelihood strategies, would contribute to ensuring long-term resilience in the study communities.

## Introduction

Shocks are “external short‐term deviations from long‐term trends, deviations that have substantial negative effects on people’s current state of well‐being, level of assets, livelihoods, or safety, or their ability to withstand future shocks” [1]. Weather shocks are extreme weather events resulted from deviations in weather or climate variable beyond the usual range of historic patterns [2]. These shocks are short-lived, abrupt, occur very rarely, lasting only from several hours to several days. Weather shocks are noticeable due to their severe impact from the usual weather pattern and cause adverse impacts on humans, infrastructure and ecosystems. Examples include very high (low) temperature, very heavy rainfall, extreme heat, flooding, drying, very high wind speed, etc. Extreme events which last for longer periods (months to years) are termed as extreme climate events [3].

*Climate refers to the average measures of weather conditions recorded over long periods (often 30 years) and the average extremes of weather conditions denote to climate shocks* [4]. The onset of climate extremes may be at sudden and short-lived (shocks), like flooding, disease outbreak, etc. or gradual and continuous (stress) like drought [5]. Climate change shocks may be expected but their consequences of particular assets, livelihoods, households, etc. may not be expected [6]. Although climate change occurs slowly over several years (30 years), it can be manifested on a seasonal or multi-seasonal scale as well [7]. *While weather observations provide information occurring on a daily basis*, *climate studies provide a sense of what to expect on average weather conditions of an area*. Despite variations in weather and climate shocks, the frequency, timing, intensity, and duration of weather events are greatly influenced by climate change and variability [3,8] and sometimes may overlap as well.

Climate change-induced shocks are climate change-related events, including rapid onset shocks (like floods, disease outbreaks, food price increase, etc.) and slow onset shocks (like drought, food price volatility, environmental degradation, etc.), which are the major livelihood threats of humanity, where underdeveloped countries are disproportionately hit by adverse effects [9]. Projections by the Intergovernmental Panel on Climate Change (IPCC) shows that the frequency and intensity of climate change-induced shocks are growing all over the world [10]. Effects of such extremes would add extra stress on human health, food security and water resources, where the rural poor are extremely susceptible and adversely impacted [10,11]. The IPCC report emphasized that disaster risk management programs should focus on reducing exposure and vulnerability while enhancing resilience to shock impacts [10].

The intensification of two huge societal trends-climate change and globalization, which amplify multifaceted and non-directional impacts have caused resilience to be acknowledged in a wide range of disciplines globally [12]. The concept of resilience stems from the Latin ‘resilire’ to denote to ‘bouncing back’ or ‘recoiling’ [13]. It was primarily applied in mechanics in 1858 referring to the capability of a material to resist a force (rigidity) as well as to absorb the force with deformation; later it was used in psychology in 1950s, in system ecology in 1973 and in social-ecological systems in the 1990s [13]. In recent understanding, resilience is conceptualized beyond engineering resilience (absorptive capacity)- “capacity to resist shock and bounce-back” and ecological resilience (adaptive capacity)- “capacity to buffer, adjust, and continue to functioning following shocks”[14]; instead it also includes the transformative capacity_” capacity to create a fundamentally new system in times of crises” [15]. In terms of climate change, absorptive capacity refers the “ability to cope with the consequences”, adaptive capacity refers the “ability to adjust changes, moderate damage and to take opportunities” [11]. Whereas, transformative capacity refers to the “ability to create a new system to make conditions attainable” [15]. The majority of academics and experts from a wide range of disciplines recognize resilience with a multiplicity of abilities, including “ability to resist and bounce back”, “ability to moderate changes and continue operating”, “ability to create a new system in times of crises” [16]. These abilities collectively denoted to the absorptive, adaptive and transformative capacities [17–19]. These capacities are termed as the core-components of resilience [17,18,20] or the three-structural elements that need to be considered during the resilience analytical framework. On the other hand, the absorptive, adaptive and transformative capacities result in persistence, incremental adjustments, and transformational responses, respectively. Moreover, resilience itself was recognized as an outcome variable in many disciplines [17].

As a result, the absorptive, adaptive and transformative terms can be considered as components, capacities or structural elements of resilience based on the context. In this study, however, resilience is conceptualized as a capacity to deal with climate change-related events; where the terms-absorptive, adaptive and transformative denote to the households’ ability to resist, adapt and transform against shock impacts. This study followed the three-capacities (absorptive, adaptive and transformative capacities) frame to explore households’ resilience to climate change-induced shocks.

The three resilience capacities can be linked depending on shock intensity. Accordingly, during minimal shock intensity (minimal impact or infrequent), it is natural that the system would block or resist it [17]. Hence, internal resistance is known as the natural characteristic of a system manifested on a daily basis where resources could block the shock enabling the system to continue functioning-highly comparable to the human immune system [21]. Absorptive capacity is especially basis to buffer short-term disturbances as well as during the beginning phase of coping of huge shocks [12]. The next adaptive resilience involving system adjustment to sustain system functioning will be exercised if the shock exceeded the absorptive capacity [22]. Adaptive capacity is “the ability of a system to adjust itself to sustain system functioning”[23]. These adjustment practices are incremental as well as learning through failure and success that adds to adaptability [24]. This capacity involves “resourcefulness-the potential to identify challenges, develop priorities, mobilize resources, to integrate experience and knowledge during crises, to plan for upcoming shock impacts” [12]. These multi-level (individuals, households, community) and incremental adjustment mechanisms for farming communities may include livelihood diversification, establishing market networks, empowering storage facilities, developing pooling among communities, introducing of shock resistance varieties, new farming practices, strengthening social networks, etc. [17,18].

In the case of high intensity (chronic leading to maximum impact due to frequency or long duration) and recurrent shocks, it may be difficult to sustain system functioning through adaptive resilience, involving transformative resilience. It is often associated to system-level changes in factors like infrastructure (example: road, communication, credit access, health facilities, etc.), governance, formal safety nets which substantially strengthen long-term resilience [18]. For instance, changing of the agrarian livelihood into resource extraction economy, ecotourism, change in resource management practices, etc. Transformative resilience may require institutional reforms, behavioral changes and technological innovations [25]. The nexus between shock intensity and the absorptive, adaptive and transformative capacities is linear where absorptive involves enhancing resistance in times of small-scale disturbance; adaptation during greater disturbance and transformative when conditions become extremely unattainable [18].

Building resilience involves intervention actions that promote the absorptive, adaptive and transformative capacities at multi-levels (individuals, households, community, region, etc.). Therefore, it would be sounder to “recognize the absorptive, adaptive and transformative capacities as different perspectives of the same reality, instead of three independent qualities that can be added” [18]. In this period of environmental uncertainty, households’ capacities need to be strengthened [12] to enable smallholder farmers to better withstand the upcoming shock impacts [26]. Because resilient households are more active to anticipate, resist, cope with and recover against shock impacts [27] as well as to sustain or improve the standard of living in the face of environmental changes [28].

Thus, this study was designed to explore the resilience of smallholder farmers to climate change-related events in Dinki watershed, central highlands of Ethiopia. Therefore, research questions, including: (i) how is the term resilience conceptualized in Dinki watershed communities? (ii) are there differences in absorptive, adaptive and transformative capacities between agro-ecological zones? And (iii) what are the determinant factors that influence households’ resilience to climate change-induced shocks in Dinki watershed? were formulated to address the desired objective.

## Materials and methods

### The study area

**Fig 1:**
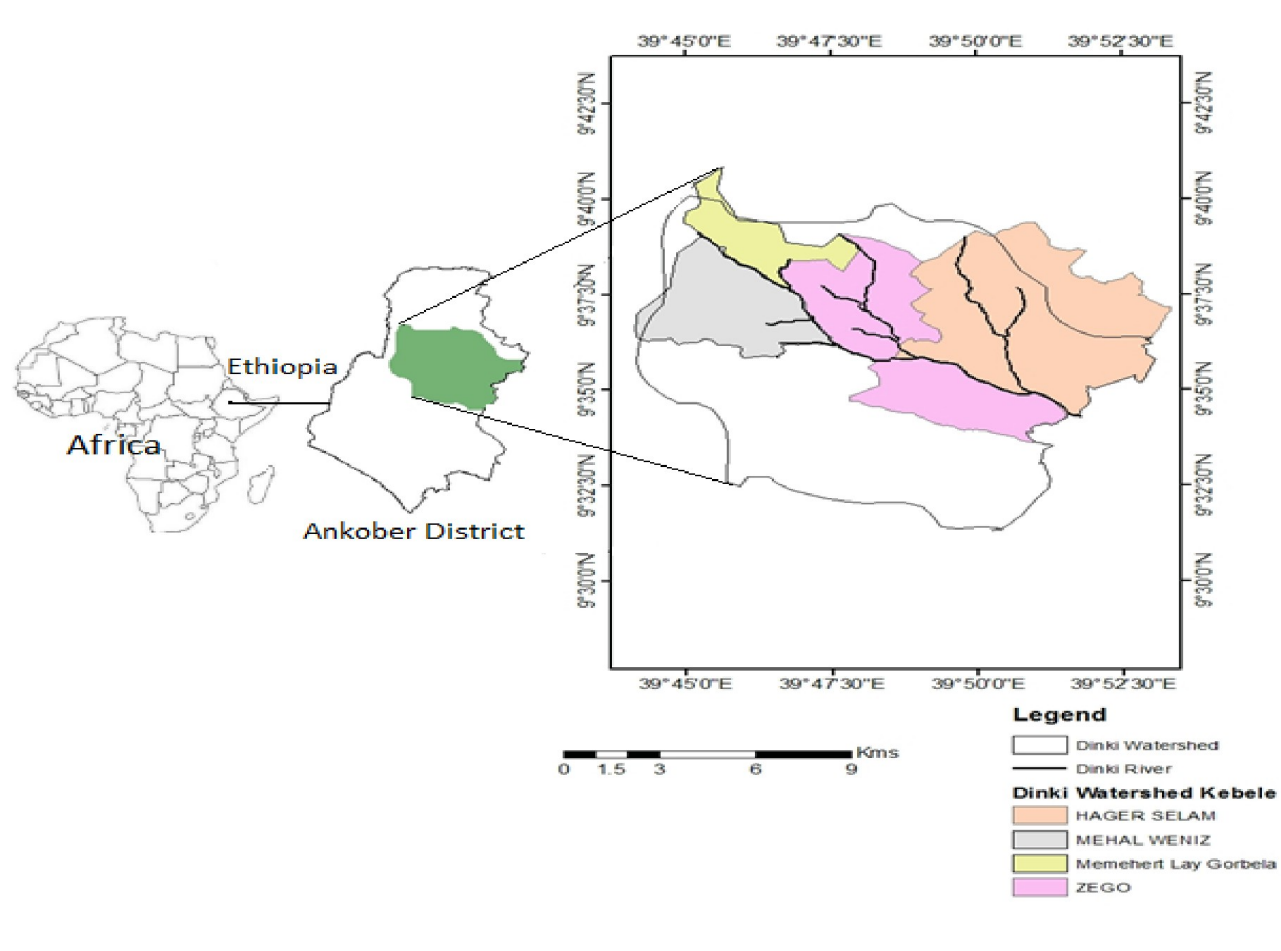
Map of the Dinki watershed, central highlands of Ethiopia.

The study area, Dinki watershed is located in Ankober district, North Shewa Zone, Amhara Regional State of Ethiopia (Fig 1). Gorobela, the city of the district, is situated at 172 km north of Addis Ababa, the capital city of Ethiopia. Ankober is located between 9^0^ 22’-9^0^ 45’ N latitude and 039^0^ 40’-039^0^ 53 E longitudes. It is found in the altitudinal range of 1300 m a. s. l. near Addis Alem to 3700 m a. s. l. at Kundi Mountain. Hills and mountains are very dominant in the district (75%); where rugged terrains and plain topography account for 17% and 8%, respectively. More than half of the district (53%) has sub-tropical (*woinadega*) climatic condition followed by tropical (*kola*); where temperate (*dega*) and cool (*wurich*) constitute 10.5 and 1.5 percent, respectively [29].

The Rainfall pattern is bimodal where some short and long-term rainy periods are recorded in March and in late June to September, respectively. A 30 year (1987–2016) of metrological data showed a mean annual rainfall of 1,179 mm; where the mean minimum and maximum monthly temperature was 6.47 and 19.99°C, respectively. The dry period often extends from October to March, but it may be extended to May. Dinki watershed is drained by a third ordered stream, Dinki River, and has a total area of 16,537 ha (Fig 1). Subsistence rain-fed agriculture integrated with livestock production is the dominant livelihood strategy for the majority of households. Traditional irrigation is rarely practiced in downstream residents to grow vegetables and perennial crops. Barley (*Hordeum vulgare*) and field pea (Pi*sum sativum*) are the dominant crops grown in the uplands, whereas *Eragrostis tef*, wheat (*Triticum vugare*), horse beans (*Vicia fabia*), etc. are prevalent in middle watershed. Whereas Zea maize and Vigna radiate (*masho*) are widely grown in the lowland agro-ecology serving as the principal livelihood options of the residents. Although cultivated land is the dominant land use types in all agro-ecological zones, there are also up areas, grass-lands, forest and water bodies.

### Data collection techniques

Data were collected through participatory rural appraisal viz: Highland, Midland and Lowland agro-climatic zones of Dinki watershed socio-ecological system, central highlands of Ethiopia. The focus group discussions and interviews were conducted during November 2017, whereas the household survey was conducted during February 2018. Prior to data collection, an ethics statement which also approved this particular study was received from the Institutional Review Board (IRB), Institute of Health, Jimma University and provided to the Ankober district administration office.

### Qualitative data collection

Three focus group discussions (FGDs) (one FGD in each agro-ecology), each comprising 8–12 participants were conducted to collect data on the households’ resilience to climate change-induced shocks. The FGD participants were from different community members like elders, religious leader, youth, women, resource management groups, school, and agriculture experts. The historic trends and socio-ecological system dynamics of the Dinki watershed during the last 50 years (from 1968–2016) were explored using “resilience-of-what?”, “resilience-to-what?” and “resilience-with-what?” questions [30]. Resilience-of-what involves identification of major constituents and spatio-temporal characterization of the area under investigation [31]. The question “resilience-to-what” explores the major disturbances of the socio-ecological system. Whereas the “resilience-with-what” searches for possible assets and livelihood options that contribute to building the absorptive, adaptive and transformative capacities [30]. The same leading questions constituting of the aforementioned concepts were also used to conduct 15 face-to-face interviews on community members selected by a snowball technique, where participants are selected upon the recommendation of others.

The focus group discussion and key informant interviews were then guided by checklists, where the leading questions were raised by the interviewer (corresponding author) and allow the discussants and the interviewee to narrate as much as they can. Information redundancy was used as insurance for information saturation and the interviewer proceeds to the next question, and so on. During the discussion and narration, one filed assistant was assigned for open note-taking. Hence, the target populations were Amharic language speakers, all the communication was carried out in Amharic. Following the individual and group interview (focus group discussion), the results were translated into English, organized and summarized through content analysis. A total time range of 25 to 30 minutes was used to complete the individual interview. However, a single focus group discussion was lasted in a range of 60 to 90 minutes and extended up to 2 hours in occasions when participants are willing to debate [32,33].

### Quantitative data collection

Following the focus group discussions and key informant interview as well as based on related literature, a paper survey questionnaire was formulated. The questions “resilience-of-what?”, “resilience-to-what?” and “resilience-with-what?” were also used in the household survey paper. A simple random sampling technique was employed to select study participants from the list of farming communities living in Dinki watershed. Selection of household members involves (i) receiving the list of N population in each agro-ecology from *respective village administrators; (ii) calculating sample size n; (iii) providing sequential number for each N household and (iv) picking of a household from the list of N population using a lottery method* [34]. The sample size determination followed the prescriptions by [35] as follows:

$$n=\frac{N*p*q*Z^{2}}{e^{2}(N-1)+Z^{2}*p*q}$$

Where: n = sample size

Z = 95% confidence interval under normal curve that is 1.96

p = 0.5 (proportion of the population to be included in the sample that is 50%)

q = None occurrence of event = 1–0.5; that is 0.5

N = size of population

e = Margin of error or degree of accuracy (acceptable error term) (0.05)

Therefore, a sample size of 294 respondents was obtained from a total of 1, 245 households. However, only 288 households, of which 82 females and 206 males properly responded and returned the questionnaire, resulting in 97.96% response rate.

The household survey was collected by enumerators selected from agricultural extension workers (development agents) in respective agro-ecology. In effect, two development agents were selected in each agro-ecology and a total of six enumerators participated in the survey. Prior to the actual survey, a copy of the consent letter introduced to the district administration office was distributed for the respective village administrator. Following this consent letter, enumerators were called for a one-day meeting to discuss on survey procedure. During the discussion, the specific data collection procedures, and general ethics of data collection were communicated. Furthermore, a pilot test was conducted on 12 households to test the suitability of the questions as well as to familiarize the enumerators with the paper survey.

### Climate Resilience Index (CRI) calculation

As resilience is a dynamic multidimensional concept, its quantification remains controversial [19]. Currently, however, proxy indicators through a composite index frame have been used to measure resilience in a wide range of literature [26,36–39]. In this study, the resilience tool developed by [36] to measure food insecurity and tested by [37] and [26] was customized to assess households’ resilience to climate change-induced shocks. The tool consists of ten major components and a household with higher average values of each component is hypothesized to be resilient to climate change-induced shocks. Stakeholders consultation (development agents, experts, and elders) and review literature [18,26,36–38] were used to select a relevant indicator and the details are presented in Table 1 below.

**Table 1:** Resilience capacities, major components, sub-components and hypothesized relationships.

Resilience capacities	Major component	Indicators	Hypothesized relationship: relatively resilient if:
Absorptive capacity	Natural disaster and climatic variability	Early warning system, preparedness, shock events during the last 12 months,	the household has access to early warning system and get prepared to shock impacts,
	Stability	Landscape position, soil fertility, SWC and awareness to climate change impacts	the majority of households’ farm land is gentle slope, good soil quality and most of it under SWC as well as if he has knowledge on climate change impacts
	Social capital	Sharing of resources and technology and membership to community-based organizations	there exist experiences of resources and technology and get involved in community-based organization
Adaptive capacity	Income and food access	Income, food insecurity and dietary diversity	a HH has an annual per capita income comparable to national average, lower HFIAS values in the range of 0–27, consumed balanced diet (≥7x carbohydrate, ≥3x protein, ≥3x vegetables and fruits in a week)
	Health	Illness score and improved toilet	lower values in the range of 0–24; has access to improved toilet open pit
	Water	Access to improved water, water sufficiency and water conflict	the HH has access to improved drinking water that can be collected within 30 minutes’ walk from home (round trip), water sufficiency during the last 12 months, no conflict due to water
	Sociodemographic status	Sex of the household head, dependency and education	male-headed households with lower dependency ratio and literate
	Assets	Asset and livestock holding, ownership to communication device and saving	With having large asset and livestock holding, access to saving and communication devices
	Livelihood strategy	Livelihood diversity, social support score, number of coping strategies and technology utilization (irrigation, improved seeds, etc.)	who have multiple income sources, higher social support score, utilize technology and apply varieties of coping strategies
Transformative capacity	Social capital	Conflict management, vertical linkage through involvement in governance	Who participate in elderly institutions, governance sustain peace and security
	Access to basic services	Access to basic public services, such as market, health services, primary school, road, credit and electricity	HH who access public services in ≤5 km or ≤1 hr walking distance from home

The CRI uses a balanced weighted technique [40] where each sub-component (indicator) contributes equally to the index. Using household-level data on these indicators, a Climate Resilience Index (CRI) was developed on an agro-ecological unit of analysis. As each major component is composed of a different number of indicators measured on different scales, the standardization considered the functional relationship between indicators and resilience [37]. In effect, two methods of standardization were employed. Indicators that are expected to have a direct relationship with resilience, such as income and food access, diversity of income sources, coping strategies, etc. were standardized using Eq (1) as:

$$Ia=\frac{Sr-Smin}{Smax-Smin}$$ (1)

Whereas indicators expected to have inversely related to resilience, such as household food insecurity and access score (HFIAs), illness score, shock events, etc. were standardized using Eq (2):

$$Ia=\frac{Smax-Sr}{Smax-Smin}$$ (2)

Where Ia is the standardized value for the indicator a, Sr is the observed (average) value of the indicator for agro-ecology r, min and max are the minimum and maximum values of the indicator across all the agro-ecology, respectively. Once each indicator has been standardized, the average value of each major component was computed using Eq 3:

$$Mr=\frac{\sum Iai}{N}$$ (3)

Where Mr is one of the ten major components for agro-ecology r, Iai is the indicator indexed by i, that make up each major component, N is the number of indicators in each major component. After values for each of the ten major components for each agro-ecology were calculated, the CRI was obtained from the weighted average of the ten components as:

$$CRIr=\frac{\sum_{p=1}^{10}WMiMri}{\sum_{p=1}^{10}WMri}$$

$$CRIr=\frac{WndcvNDCVr+WifaiIFAr+WhHr+WwWr+WsbSBr+WsdpSDPr+WlvsLVSr+WasASr+WscSCr+WabsABSr}{Wndcv+Wifa+Wh+Ww+Wsb+Wsdp+Wlvs+Wa+Wsc+Wabs}$$ (4)

Where CRIr is the Climate Resilience Index for each agro-ecological zone, Mri = the number of indicators of the major component, WMi = weight of major component i, NDCV = natural disaster and climate variability, IFA = income and food access, H = health, W = water, Sb = stability, SDP = sociodemographic profile, LVS = livelihood strategy, A = assets, SC = social capital, ABS = access to basic services.

Similarly, the analytical framework proposed by [18], [17] and [19] suggest absorptive, adaptive and transformative capacities as the three core components of resilience deem to sound for resilience analysis. In this sense, the FAO’s Resilience Index Measurement and Analysis (RIMA) indicators [36] were aggregated by equal weighting approach and subsumed into the three resilience components to capture households’ resilience to climate change-induced shocks [12,17,18,20]. Where resilience is the function of the three core components expressed as:

CRIr=f(ABCr,ACr,TCr) (5)

Where CRIr is the climate resilience index for the agro-ecology

ABCr is the absorptive capacity for the agro-ecology

ADCr is the adaptive capacity for the agro-ecology

TCr is the transformative capacity for the agro-ecology

The absorptive capacity is the ability of a socio-ecological system to prepare for, mitigate with or prevent negative impacts through coping strategies in order to preserve and restore basic structures and functions [17]. The index was computed based on the awareness level of households to climate change-induced shocks, access to the early warning system, preparedness, stability and social capital like sharing of resources, technology, and membership to community-based organizations [18].

Adaptive capacity is the ability of a system to adjust impacts to moderate potential damage, to take advantage of an opportunity, so that it continues functioning without significant change in system structures [11]. Examples include, livelihood diversification, introducing drought-resistant seed varieties (like growing of *Vigna radiate* or mung bean or *masho in Amharic*). In effect, income and food access, assets, livelihood diversification strategies, etc. were placed under adaptive capacity [18,20]. Transformative capacity is the ability to create an enabling new system in times of crises [15]. It is often associated with system-level changes in factors like infrastructure (example: road, communication, credit access, health facilities, etc.), governance, formal safety nets which substantially strengthen long-term resilience. As a result, access to basic services, social capital like conflict management mechanisms and vertical linkages were captured under transformative capacity [18,20]. Therefore, indicators presented in Eq (5) were aggregated into respective resilience capacities to generate the climate resilience index (CRI) as follows:

$$CRIr=\frac{WabcABCr+WadcADCr+WtcTCr}{ABCr+ADCr+TCr}$$ (6)

Where CRIr is the resilience index for the agro-ecology r; Wabc, wadc and wtc are the weight of absorptive, adaptive and transformative capacities, respectively; ABCr, ADCr and TCr are the number of indicators in absorptive, adaptive and transformative capacities in each agro-ecological zone, respectively.

On the other hand, in order to enable inter-household comparison as well as to explore most relevant indicators for intervention action in enhancing resilience to climate change-induced shocks, methods other than balanced weighted approach are more useful. In this aspect, the factorial analysis through Principal Component Analysis (PCA) is one of the multivariate techniques applied to both data reduction and identification of the dominant factors for households’ resilience [20,41]. Mathematically, a principal component is a multivariate technique that used to extract an initial set of n correlated variables into few uncorrelated (orthogonal) linear combinations of factors that capture the common information most successfully called principal component [42]. Intuitively, the principal component is a linear weighted index of all the variables presented in an ordered fashion, where the first principal component (PC1) explains the largest amount of variation in the original data. The second principal component (PC2) is entirely uncorrelated to the first principal component and explains additional but less variation than the PC1 [43]. Thus, components are completely uncorrelated to the previous components and each component contains an additional but smaller proportion of variation than the original variables [42].

In this study, a two-stage procedure was used to estimate households’ resilience index. During the first stage, eight resilience blocks were estimated using principal component analysis based on 30 indicators. These resilience blocks include climate change and variability (CCV), income and food access (IFA), access to water and health facilities (AWH), assets (A), adaptive capacity (AC), social capital (SC), access to basic services (ABS) and stability (S). Based on the factor loading of each indicator, the resilience index of each individual household was computed using the PCA following the equation as:

RIa=f1x(a*1−a1)/(S1)+f2x(a*2−a2)/(S2)+⋯+fNx(a*N−aN)/(SN)

Where RI_a_ is the resilience index for the household a; *f*_1_ is the component loading generated by the PCA for the first variable; a*_1_ is the measured value of the variable 1; a_1_ and S_1_ are the mean and standard deviation, respectively of the first variable overall the household.

During the second procedure, the latent variables (resilience blocks) estimated during the first stage became covariates to create the whole resilience index. Simultaneously, the resilience index calculated for each household was considered as a dependent variable for further analysis in multiple regression [44]. Hence, a multiple linear regression model was used to predict the resilience index (dependent variable) based on a set of independent variables using the equation as follows:

Y=ɑ+B1X1+B2X2+⋯+BkXk+e

Where, Y is the resilience index (dependent variable); a is the intercept; bs and Xs are the coefficients and the set of predictors, respectively [43]. Thirty-four explanatory variables were included in the regression model; of which 27 indicators statistically predicted the resilience (S6 Table). The bivariate correlations of variables were presented in the supportive information section (S2–S5 Tables).

## Results and discussion

### Determinants of resilience as estimated by PCA

The result of the principal component analysis based on the resilience blocks generated four possible components contributing to a cumulative variance of 82.99% using an eigenvalue cut-off of 1.0. The four factors explained 42.24%, 14.98%, 13.55% and 12.22% of variations, respectively. Accordingly, except climate change and variability, all the remaining components were positively and heavily correlated with resilience. As a whole, IFA on factor 1 was the most important component contributing to resilience; whereas AC, ABS and SC were also vital components supporting resilience to climate change-induced shocks (Table 2). As a result, in Dinki watershed socio-ecological system, integrated management of all the resilience blocks contributes to enhancing households’ resilience to climate change-induced shocks. However, dimensions like income and food access, adaptive capacity, access to basic services and social capital should get great concern and priorities to enhance resilience to shock impacts.

**Table 2:** Loadings of resilience blocks on the first four principal components, uniqueness and proportion of variance explained by each component.

Variable	Component				
	Factor1	Factor2	Factor3	Factor4	uniqueness
Income and food access	**0.857**				0.142
Access to water and health facilities	**0.804**				0.284
Asset	**0.791**				0.172
Climate change and variability	**-0.695**				0.300
Adaptive capacity		**0.926**			0.12
Stability		**0.846**			0.179
Access to basic services			**0.935**		0.119
Social capital				**0.972**	0.044
Eigenvalue	3.379	1.198	1.084	0.978	
%of variance	42.24	14.98	13.55	12.22	
Cumulative %variance explained	42.24	57.22	70.77	82.99	
N 288					
KMO 0.738					
Sphericity test Σ^2^(28) = 948.13, p<0.001					

Source: Authors’ own construction from household survey (February 2018)

Notes: The principal component analysis result has passed the diagnostic assumptions such as multicollinearity, uniqueness and sphericity tests.

### Determinant of resilience as estimated by multiple regression analysis

#### Exposure to shock events, access to early warning system, social capital and stability (farmland location, soil fertility, and management)

These are indicators of the absorptive capacity (Table 1; S1 Table). It is hypothesized that households having exposure to shock events, those whose most of their farmlands are infertile and located in steep slopes are more vulnerable to climate change-induced shocks like soil erosion, landslides, food insecurity, etc. These households are more likely to be poorly resilient to climate change-induced shocks due to their higher sensitivity and limited livelihood options. In this study, households who experience shock events have -0.459 point, indicating for a household exposed to one shock event, the likelihood of recovery would decline by 46% than households who have not exposed to any shock. Likewise, household who have a higher proportion of infertile and steep slope (inconvenient to farm) farmlands have -0.24 and -18.78 points, respectively. On the other hand, households who have access to the early warning system, awareness on climate change impacts, experience sharing of resources and technology during shocks events, having a higher proportion of farmland under soil and water conservation measures have 0.07, 0.18. 0.12 and 0.13 points of likelihoods to become more resilient to climate change-induced shocks (Table 3). This suggests that these parameters contribute to mitigate with, adapt to and quickly recovery against shock impacts.

**Table 3:** Resilience categories and factors influencing households’ resilience to climate change-induced shocks in Dinki watershed socio-ecological system.

Factors	Resilience category		
	Poor resilience or likely highly vulnerable	Moderate resilience	Highly resilience or likely less vulnerable
Recovery time to normal agricultural operation	Bounce back in more than two agricultural seasons	Bounce back within one-two agricultural seasons	Bounce back within one agricultural season
Farm plot size (ha)	≤one	one-two	≥two
Livestock holding (TLU)	≤one	one-two	>two
Social protection (resource, labor, group)	Poor social protection	Moderate social protection	Strong social protection
Diversity of income sources	Solely rely on rainfed crop farming	a minimum of 2 income sources	2–3 income sources at least some period of the year
Ecological stability (location, fertility and soil and water conservation (SWC) measures)	≥50% of their land is in steep slope and/or infertile and or located at the edge of river bank or no SWC or ≤25% SWC cover	25–50% of their land in steep slope and/or infertile and or located in near river bank or 25–50% SWC cover	≤25% of their land in steep slope and/or infertile; most of their lands are located a bit distant from river banks or ≥50% SWC cover
Infrastructure	Access to major public services in ≥2-hour walk	Access to major public services in 1-2-hour walk	Access to major public services in ≤1-hour walk

The result of the household survey was also complemented by the key informant and FGD participants. They presented the socio-ecological dynamics of Dinki watershed during the last 50 (1968–2016) years. Accordingly, land degradation, change of state, food insecurity, disease manifestation, market fluctuation and erosion of indigenous knowledge and practices were identified as key disturbances during the study period. Of which, climate change and variability, land degradation and erosion of indigenous knowledge and practices were identified as the three top changes affecting the study community using pairwise ranking. In this connection, the participants perceive resilience as a state of recovery against climate change-induced shocks without significant help from external institutions. However, the effects of climate change-induced shocks and consecutive rate of recovery are not uniform across households. In effect, households in Dinki watershed socio-ecological system were classified into poor resilient, moderately resilient and resilient based on their recovery time to climate change-induced shocks. Such classification was also reported in other parts of Ethiopia [20]. Key determinants of resilience and major features of each resilience category are presented in Table 3 below.

Discussants reported that that household whose farmlands are located in steep slopes and near to river banks is highly vulnerable to soil erosion and flooding impacts. Likewise, land fertility is also reported as a principal factor influencing households’ productivity and wealth status. Accordingly, households whose farmlands are in gentle slope and with better soil fertility are better off in production and are relatively resilient to shock impacts than their counterparts. Discussants and key informants identified that land resource management practices mainly through soil and water conservation contribute to influencing households’ resilience to climate change-induced shocks like soil erosion. In effect, households who experience intensive soil and water conservation measures are less likely impacted by erosion and more likely to recover quickly against the adverse impacts of erosion. Thus, poorly resilient households are those whose most of their lands are located in steep slopes, proximate to river banks, with infertile and minimal soil and water conservation practices and thereby less resilient to shock impacts.

In agreement with the current result, studies state that climate change has a negative impact on households’ resilience. In effect, improving access to assets, early warning system and social safety nets could contribute to mitigate with and adapt to the effects of climatic extremes [45]. Moreover, in Ethiopian farming community, the indigenous knowledge through weather forecasting (example: cloud color, wind direction, sound of birds, etc.) contributes to decide on farming, harvesting and associated daily activities [41]. Others highlight that land location and fertility are critical to determine farm productivity. Accordingly, households with improved land fertility are better off in farm production and more resilient to shocks [20,46]. For instance, in undulating terrain areas like in the study area, farming and settlements expand to steep slopes and forested areas, resulting in accelerated soil erosion as well as erosion of households’ resilience to shock impacts [41]. As a result, watershed management through afforestation, enclosure and agro-forestry practices are reported to contribute to enhance households’ resilience to climate change-induced shocks [41,46].

#### Livelihood diversity, asset holding, social capital and sociodemographic profile

The majorities of these are indicators of adaptive capacity (Table 1; S1 Table). It is hypothesized that households who have multiple income sources, large asset holding and strong social capital become more resilient to climate change-induced shocks than their counterparts. In this study the coefficient of income diversity was 0.14, indicating the probability of increasing recovery following shock impacts by 14% if the household added one enterprise than normal livelihood system. Likewise, the coefficient of the number of coping strategies was 0.09, indicating 9% of faster recovery if the household experience one more coping strategy to shock impacts than the normal system (Table 4). Positive and direct association of diversity of income sources with resilience was also reported by various studies [38,41,46].

**Table 4:** Multiple linear regression analysis results on determinants of livelihood resilience to climate change-induced shocks.

Explanatory variables	Regression coefficient ±standard errors (SE) of each agro-ecology			
	Highland	Midland	Lowland	Aggregate data
	Coefficient ±SE	Coefficient ±SE	Coefficient ±SE	Coefficient ±SE
Family size	-0.87*±0.34			-6.78*** ±1.22
Age of the household head				0.09*** ±0.02
Sex of the household head		0.09*±0.05	0.17*±0.07	0.12*** ±0.03
Farm size	0.05*± 0.02	0.12**±0.04	0.19**0.07	0.16*** ±0.02
Livestock holding	0.23***± 0.03	0.26***±0.05	0.23** ±0.07	0.22*** ±0.03
Access to communication device	0.23***±0.04	0.29***±0.06		0.12***±0.03
Access to early warning system	0.07*** ±0.02	0.14**±0.05		0.09* ±0.02
Sharing of resources and technology	0.12**± 0.04			0.12*** ±0.02
Vertical linkage (participation in governance)		0.14** ±0.04		
Exposure to shock events		-4.59* ±2.28		-18.13***±3.8
Injury/death due to shocks	-5.81***±01.26		-11.90*** ±2.57	
Awareness to climate change impacts	0.18*** ± 0.02	0.29***± 0.05		
Membership to community-based organizations			0.17* ±0.07	0.06* ±0.03
Water sufficiency	0.06*±0.02	0.27*** ±.05		
Water conflict	0.10*** ±0.02	0.15** ±.04		
Social support score	0.06** ±0.02			0.06* ±0.03
Land under soil and water conservation	0.13** ±0.04			0.19*** ±0.03
Proportion of steep slope farm land (topography)	-0.24***±0.04			
Proportion of infertile soil		-18.78** ±5.31		
Diversity of income sources	0.14** ±0.04	0.19**±.06		0.07* ±0.04
Growing of perennial crops			0.14* ±.06	
Number of coping strategies	0.09**±0.03		0.31** ±0.09	0.16*** ±0.03
Access to local market	0.15***±0.03		0.29*** ±0.07	0.12*** ±0.02
Access to credit	0.06* ±0.02			0.08*** ±0.02
Access to health centers		0.14** ±0.04		
Access to road network	0.07** ±0.02	0.23** ±0.09		0.17*** ±0.03
Access to primary school		0.19 **± 0.06		
(Constant)	13.54*** ±2.69	14.42*** ±3.97	18.42*** ±4.17	64.08***±8.96
N	95	98	95	288
R-squared	0.987	0.929	0.819	0.927
Adj. R-squared	0.984	0.916	0.802	0.922

*p<0.05

**p<0.01

***p<0.001

Source: Authors’ own construction from household survey (February 2018)

Notes: The multiple linear regression model result has passed the diagnostic assumptions such as multicollinearity, normality, linearity and Durbin-Watson tests.

Discussants and key informants disclosed that households who experience multiple livelihood options have more assets and improved living standards. In this aspect, female discussants stated that small-scale irrigation, home garden, and small-scale trading are essential in supporting the income-generating ability of women and youth. Two female informants in *Mehal-Wonz* (Highland) and *Zego* (Midland) sites disclosed that selling of alcohol, locally termed as *tela* and *areki* have substantial contribution in improving their standard of living, especially in fulfilling children’s demands of clothing and stationery materials. In general, households with a diversity of income sources are less vulnerable; instead more likely quickly recover against climate change-induced shocks than who solely depend on a single source of income. In agreement with this finding, studies state that income diversification is a strategy to improve the income-generating ability of women in rural households [47]. As a result, livelihood diversification is attributed with both coping strategies to risks in times of hazard events and a means of livelihood development in conducive economic settings [48].

Discussant added that access to and size of farmland is the principal factor that determines household’ resilience to shock impacts. They stressed that land ownership is a priority for the farming community for long-term decision and soil fertility management options. Accordingly, landless households are less likely to work on natural resource management practices even may amplify environmental degradation through overexploitation. Whereas, households with large farm sizes are more likely to invest on land and soil fertility management works, diversify income sources (crop-livestock integration, polyculture, agroforestry, etc.) and more likely to bounce back quickly against shock impacts. In agreement with this finding, studies state that landlessness and small land holding are determinant factors causing land degradation and resilience erosion [20]. Besides, a study in central Ethiopia discloses that natural resource management practices, which in turn determined by farm size, among others, are strategies for rural communities to enhance their resilience to shock [46].

Livestock holding is argued to signify wealth and dignity in rural Ethiopia. Discussants disclosed that livestock ownership is a determining factor for household livelihood and sustainability; as households having domestic animals are more likely to enhance and diversify income sources. However, the number and diversity of animals critically influence their economic returns. Accordingly, Oxen ownership is a priority for every farmer to secure his agricultural production. The next priority is reported to have milking cow to sustain livestock production and dietary diversity. Depending on the agro-ecology and households’ choice, having transportation animals, such as donkey/horse/mule/camel would be the next interest. Because, in areas with limited car access, like the study area, humans and materials, including agricultural inputs (fertilizer, improved seeds, pesticides), market inputs and related commodities are transported through these animals; markedly supporting livelihood options, asset accumulation and recovery to shocks. As a result, households with more than two TLU (a minimum of 2 Oxen or 1 Ox + 1 cow) are more likely to be more resilient to climate change-induced shocks. In line with this finding, a study in other parts of Ethiopia states that asset holding, including land and livestock unit, is determinant to diversify income sources, improves income and critical for the households’ resilience to food insecurity [49]. Participants added that social networking is a determinant factor for mankind to share labor and resources, manage disputes as well as to mitigate with, adapt to and quickly recover against shock impacts. See also [18,20,41,50].

On the other hand, the family size was negatively correlated with resilience, where for every one individual added to the household, the likelihood of the household resilience drops by 68%. However, for every one-year increase in the life of the household head, the probability of enhancing resilience was increased by 9%. In terms of gender, for every one male-headed household added in the community, the likelihood of increasing resilience was 12% (Table 4). Similarly, resilience is reported to have an inverse relation with large family size [41] and with female-headed households [37,38].

#### Access to basic services and vertical linkages

These are indicators of the transformative capacity (Table 1; S1 Table). It is hypothesized that households who have access to basic public services as well as who actively participate in the governance system will have access to information, create positive social bonding and relatively more resilient than their counterparts. In this study, the coefficients of market, credit and road network in the aggregated points were 0.12, 0.08 and 0.17, respectively. This indicates that for a HH having one kilometer nearest access to market, credit and road facilities, the probability of recovery following shock impacts could increase by 12%, 8%, and 17%, respectively. Similarly, the coefficient of vertical linkage was 0.14, indicating for every one household head participated in the governance system, the probability to enhance resilience would increase by 14% (Table 4). In agreement with the current study, participation in local institutions has also reported contributing positive relation in building resilience to climate change-induced shock [41].

Key informants noted that infrastructure, mainly road and market are a basis for further societal developments. In this aspect, access to basic infrastructures is minimal where only 18.06 and 55.56% of households access all-weather road and market within five km distance, respectively, making the study communities isolated from market centers. In agreement with this finding, studies state that underdeveloped infrastructure is a driving cause for insufficient access to public services, minimal market integration and little returns on investments [51]. Hence, geographically isolated communities who live distant from the main road and local market experience minimal access to inputs, market exchange, information as well as livelihood diversification opportunities [46,52]. Likewise, [53] argue that access to basic infrastructure is determinant in promoting households’ resilience to shocks by enhancing their access to assets. Access to credit services was also minimal where only 59.38% of households access to credit facilities in their proximity. Studies state that insufficient physical structures significantly limit access to basic services like health and credit facilities, contributing socioeconomic marginalization [54]. In effect, the lack of access to cash needs during crises is a major factor limiting households’ resilience to climate change-induced shocks [46].

#### Households’ resilience as measured by Climate Resilience Index and resilience capacities

The livelihood resilience analysis through the three-capacities and Climate Resilience Index showed relatively comparable results. Accordingly, the highland is better off in sociodemographic profile, water, and health; the midland is better off in exposure to natural disaster and livelihood strategies and the lowland is better off in income and food access, asset, stability, social capitals and access to basic services (S1 Table; Table 5).

**Table 5:** Indexed major components, core-capacities and overall Livelihood Resilience Index (LRI) of Dinki watershed socio-ecological system. (NDCV = Natural Disaster and Climate Variability; IFA = Income and Food Access; SDP = Sociodemographic Profile; LVS = Livelihood Diversity and ABS = Access to Basic Services).

Resilience capacities	Major components	Agro-ecology					
		Highland		Midland		Lowland	
		Component value	Resilience score	Component value	Resilience score	Component value	Resilience score
Absorptive	NDCV	0.472		0.657		0.503	
	Stability	0.45	**0.448**	0.414	**0.517**	0.412	**0.520**
	Social capital	0.404		0.419		0.693	
Adaptive capacity	IFA	0.412		0.491		0.516	
	Health	0.46	**0.436**	0.416	**0.495**	0.399	**0.417**
	Water	0.544		0.465		0.361	
	SDP	0.569		0.455		0.459	
	Assets	0.288		0.31		0.371	
	LVS	0.343		0.444		0.385	
Transformative capacity	Social capital	0.505		0.499		0.542	
	ABS	0.35	**0.389**	0.327	**0.37**	0.355	**0.402**
Overall LRI							**0.444**

**Fig 2:**
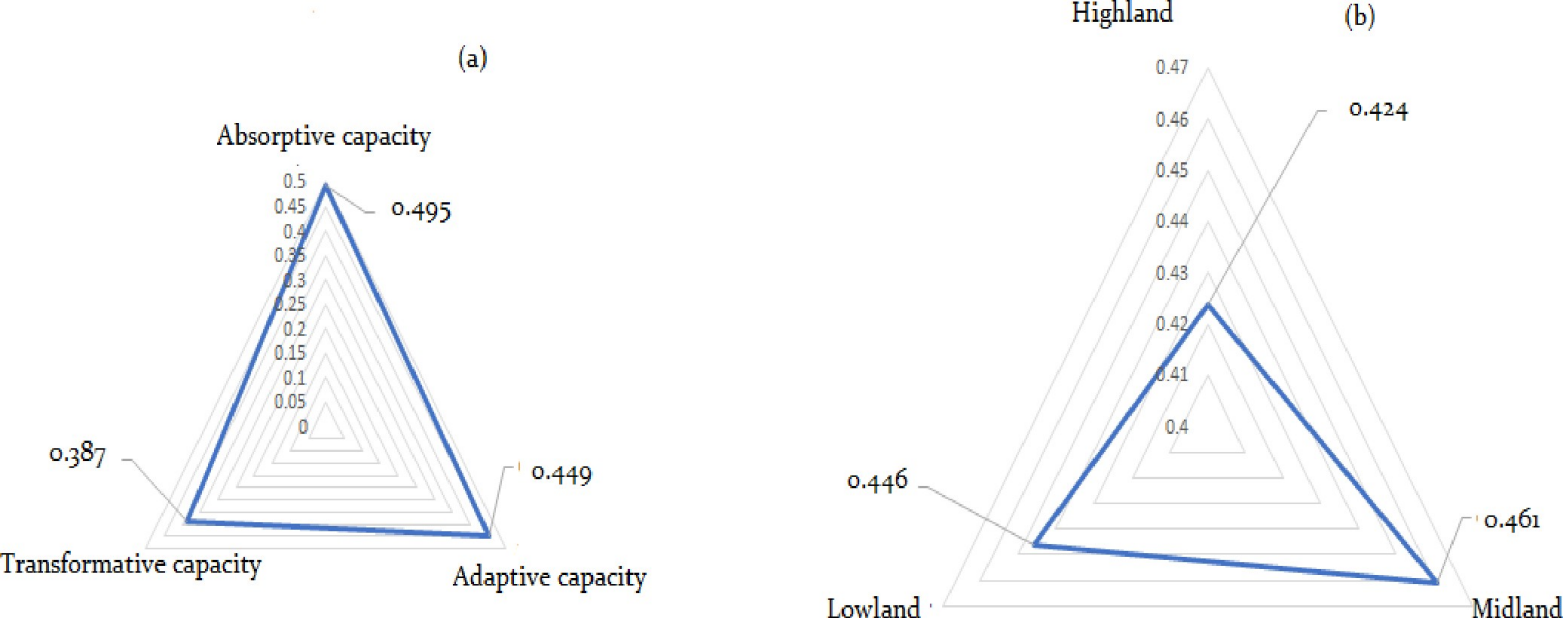
The core-resilience capacities (a) and resilience status of agro-ecological zones (b).

The livelihood resilience analysis through resilience capacities more clearly differentiated the agro-ecological zones in terms of their absorptive, adaptive and transformative capacities. In effect, the leading contributing factor to the resilience of Dinki watershed socio-ecological system to climate change-induced shocks was observed to be absorptive capacity with a mean index value of 0.495 followed by adaptive capacity with a mean index value of 0.449 (Fig 2A). In terms of agro-ecology, the Midland was found to be relatively more resilient to climatic shocks with a mean index value of 0.461(Fig 2B).

Relatively higher score of absorptive capacity in the lowland agro-ecology is evident by the fact that its exposure to recurrent climate change-induced shocks might have enabled residents to acquire more knowledge and get prepared for future likely shocks. Besides, large farm and livestock holding, social capital (CBOs, SSS, sharing of resources and technology) as well as coping strategies (in economic and management options) might have enabled lowland residents to better absorb shocks compared to the highland and midland agro-ecological zones.

In line with this study, [41] disclose that households in Ethiopian lowland areas often have quick access to climate change information and early warning system contributing to their improved preparedness compared to other climatic zones. Other studies argue that large farm and livestock holding enable households to spread risks through income diversification and asset accumulation opportunities [49]. Moreover, [18] disclose that households’ ability to diversify financial capital, natural capital and social capital, among others, reduces their vulnerability, whilst enhancing their absorptive and adaptive capacities to properly respond to changing conditions [18].

On the other hand, the resilience score in terms of adaptive capacity was higher in the midland followed by the highland. It might be due to the fact that improved livelihood diversification practices (trade, irrigation, tree garden), technology utilization (improved seed and fertilizer) and improved access to credit might have enabled the midland and highland residents to better adapt climate change-induced shocks. Moreover, informal institutions like *idir* and *equib* are basic economic leverage contributing households to better adapt to shock impacts. In agreement with this finding, studies state that livelihood diversification, information exchange, and economic leverage institutions contribute to enhance households’ adaptive capacity to shock impacts [18].

Although the mean resilience score in terms of transformative capacity (0.387) is lower to other resilience scores (Fig 2A), the lowland showed the highest transformative capacity (0.402) than the other agro-ecological zones (Table 5). A relatively higher proportion of households who access market in their proximity coupled with higher social capital (transformative) scores through conflict management and vertical linkages in the Lowland and Highland might have contributed to higher transformative capacity in these agro-ecological zones. In this aspect, disputes over access to water, pasture and related land resources are repeatedly reported as major sources of conflict in the study community. As a result, conflict management options through elders’ institutions might have contributed to building peace and security among the study communities.

In agreement with this finding, studies state that managing conflict ensures information exchange and market linkage with other communities leading to knowledge sharing. Besides, the participation of community members in decision options facilitates information dissemination, access to basic assets during crises and enhance transformative capacity through institutional reforms [18]. Furthermore, conflict management through customary laws is recognized as plausible options to sustain social capital among Africans [55]. These institutions are participatory, easily accessible and sustainable in keeping peace and thereby resilience [18].

**Fig 3:**
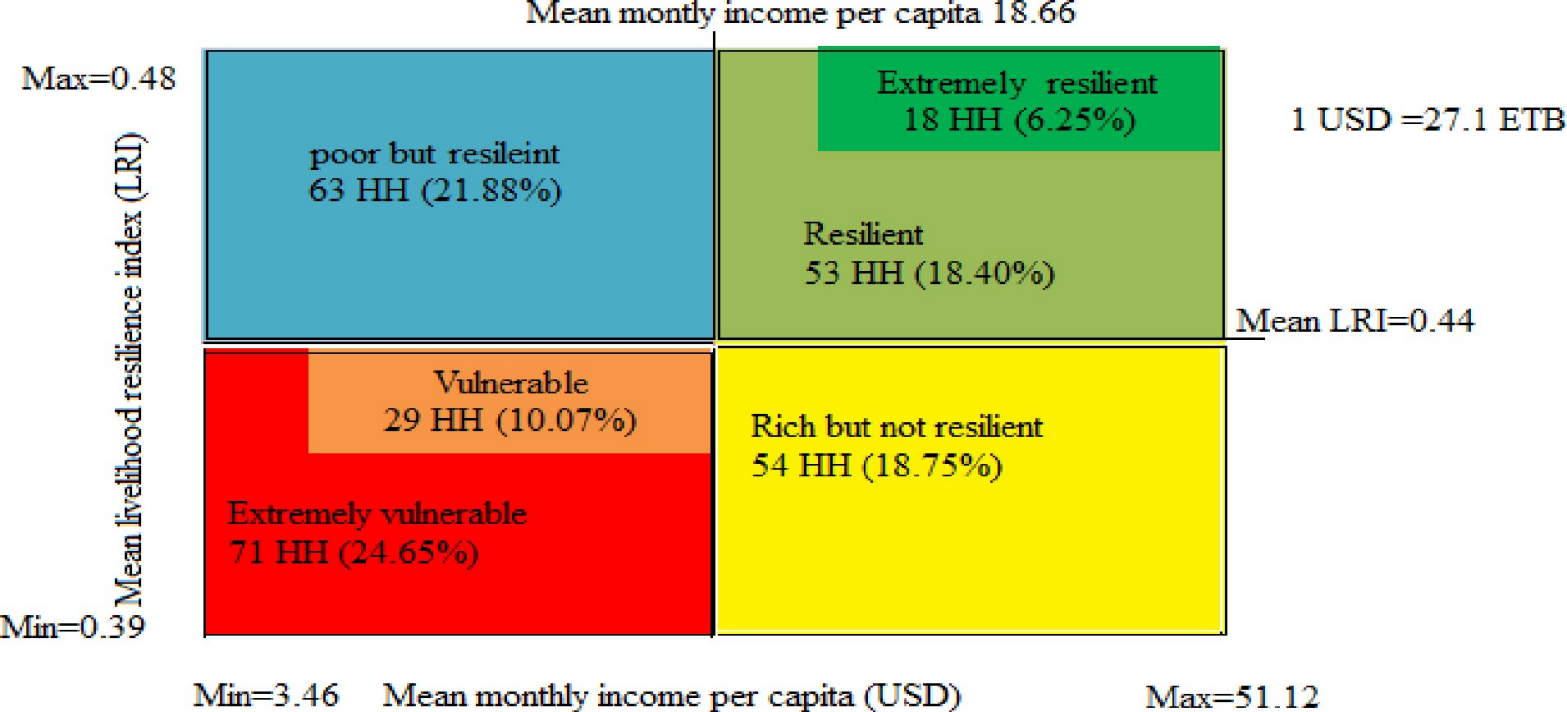
Resilience typologies by households’ monthly income.

Furthermore, households’ resilience capacity was graphically presented in four-quadrant charts following the [56] and [20]. The graph was established based on households’ income per capita and mean LRI values drawn on x and y-axes, respectively. Accordingly, based on the mean LRI value (0.44), households falling above the mean were poor but resilient, resilient and extremely resilient. Whereas rich but not resilient, vulnerable and extremely vulnerable households were presented below the mean. Likewise, based on the mean monthly income (18.66 per month or 0.622 USD per day), households falling to the right of the mean include rich but not resilient, resilient and extremely resilient. Whereas households who were poor but resilient, vulnerable and extremely vulnerable were presented in the left of the mean (Fig 3).

The average daily income value is far below the poverty line of sub-Saharan Africa, indicating the poverty level of the study communities. Moreover, even with this minimal cutoff, more than half of the households (56.59%) were vulnerable to poverty (Fig 3). Factors, such as small asset holdings coupled with underdeveloped infrastructure might have limited their adaptive capacities signified by poor diversification practices while amplifying their vulnerability.

In this study, a considerable proportion (32.29%) of households own less than one hectare of land, nearly half (47.22%) of the study communities have less than two livestock unit and the overall infrastructure is underdeveloped (S1 Table). Moreover, the majority of households (94.79%) experience a single income-dominated livelihood option making them vulnerable to climate change-induced shocks. In agreement with this finding, studies disclose that land and livestock are two of the most known financial assets in farming communities of Ethiopia critically determining their wealth status [49]. Unlike to the Chayanov’s theory of consumption-labor-balance, the results in this study showed that households with large asset holdings have more access to basic households’ capitals (human, financial, physical, natural, social capitals) and tend to invest more on their farms *(*http://era.anthropology.ac.uk/Era_Resources/Era/Peasants/theory07.html).

Thus, poor households are often with small land size, few livestock units, and minimal livelihood options as well as are with minimal access to key household assets (natural, physical, human and social capitals) to diversify livelihoods and to empower their adaptive capacity [57]. However, poor people are not necessarily vulnerable if have access to communication, infrastructure and support systems [58]

## Conclusion

In this study, the Climate Resilience Index based on resilience capacities frame has differentiated the agro-ecological zones in terms of their absorptive, adaptive and transformative capacities. Besides, the principal component analysis has generated major factors that contribute to households’ resilience to climate change-induced shocks. Likewise, the multiple regression analysis identified the determinants of resilience. The methods presented a detail description of factors contributing to households’ resilience to shock impacts. As a result, access to and use of livelihood resources, such as farmlands, livestock, livelihood diversification, infrastructure, as well as social capital and ecological stability are identified to influence households’ resilience to climate change-induced shocks. However, it might be due to their exposure to recurrent shocks coupled with constrained adaptive capacities like limited diversification practices, poor access to infrastructure, underdeveloped social capital, among others, the mean resilience score of the study communities is minimal. Similarly, although improved absorptive capacity through early warning system, social protection, climate change information, etc. contributes to prepare, anticipate and cope with shock impacts, it is equally important to strengthen both the adaptive (adjustment strategies) and transformative (system-level change) capacities to ensure long-term resilience in the study communities.

## Supporting information

S1 Table Indexed major components, sub-components and overall CRI of Dinki watershed socio-ecological system. (DOCX)

S2 Table Bivariate correlation of variables in the highland agro-ecology (RI = resilience index, CC = climate change, IncomeD = income diversity, SSS = social support score, SWC = soil and water conservation, EWS = early warning system, wSuff = water sufficiency, wConflict = water conflict). (DOCX)

S3 Table Bivariate correlation of variables in the midland agro-ecology (partGonce = participation in governance). (DOCX)

S4 Table Bivariate correlation of variables in the lowland agro-ecology (CBOs = community-based organizations). (DOCX)

S5 Table Bivariate correlation of variables in the combined data. (DOCX)

S6 Table Variables, weighted and indexed values included in the study. (XLSX)
