# Supplementary material for: Exploring households’ resilience to climate change-induced shocks using Climate Resilience Index in Dinki watershed, central highlands of Ethiopia
Source: PLoS One. 2019 Jul 9;14(7):e0219393. doi: 10.1371/journal.pone.0219393 (PMC6615616; doi:10.1371/journal.pone.0219393)
Supplement: S1 Table — (DOCX) [file pone.0219393.s001.docx]

S 1 Table. Indexed major components, sub-components and overall CRI of Dinki watershed socio-ecological system

| Major component | Indicator | Highland | | Midland | | Lowland | |
| --- | --- | --- | --- | --- | --- | --- | --- |
|  |  | Value of indicator | value of major component | Value of indicator | value of major component | Value of indicator | value of major component |
| Natural disaster and climatic variability | %HH reporting >1 Environmental shock during the last 12 months | 0.147 | 0.472 | 0.633 | 0.657 | 0.168 | 0.503 |
|  | %HH reporting >1 Socioeconomic shock during the last 12 months | 0.14 |  | 0.694 |  | 0.126 |  |
|  | %HH with injury/death due to shocks during the last 12 months | 0.453 |  | 0.776 |  | 0.453 |  |
|  | %HH having early warning system | 0.86 |  | 0.479 |  | 0.905 |  |
|  | %HH prepared to future likely CC impacts | 0.76 |  | 0.704 |  | 0.863 |  |
| Stability | %unsuitable land slopes(topography) | 0.472 | 0.450 | 0.588 | 0.4145 | 0.613 | 0.412 |
|  | %infertile soil | 0.518 |  | 0.518 |  | 0.482 |  |
|  | %land under SWC | 0.632 |  | 0.172 |  | 0.073 |  |
|  | perception to CC impacts | 0.179 |  | 0.38 |  | 0.48 |  |
| Social capital | resources sharing b/n HHs | 0.54 | 0.404 | 0.56 | 0.419 | 0.947 | 0.693 |
|  | technology sharing b/n comm | 0.52 |  | 0.52 |  | 0.9474 |  |
|  | membership to CBOs | 0.152 |  | 0.1777 |  | 0.184 |  |
| Income and food access | Annual per capita income | 0.274 | 0.412 | 0.325 | 0.491 | 0.358 | 0.516 |
|  | HFIAS | 0.605 |  | 0.711 |  | 0.675 |  |
|  | Dietary diversity | 0.358 |  | 0.439 |  | 0.516 |  |
| Health | Illness score | 0.801 | 0.460 | 0.7912 | 0.416 | 0.689 | 0.399 |
|  | Improved toilet | 0.12 |  | 0.04 |  | 0.11 |  |
| Water | Improved water | 0.421 | 0.544 | 0.429 | 0.465 | 0.453 | 0.361 |
|  | Water sufficiency | 0.526 |  | 0.327 |  | 0.189 |  |
|  | Water conflict | 0.684 |  | 0.64 |  | 0.44 |  |
| Sociodemographic status | %female headed households | 0.906 | 0.569 | 0.663 | 0.455 | 0.747 | 0.459 |
|  | Age of household head | 0.362 |  | 0.415 |  | 0.351 |  |
|  | Dependency ratio | 0.78 |  | 0.47 |  | 0.475 |  |
|  | %Literate HH heads | 0.523 |  | 0.418 |  | 0.421 |  |
|  | Family size | 0.275 |  | 0.31 |  | 0.3 |  |
| Assets | Farm size | 0.4027 | 0.288 | 0.473 | 0.310 | 0.541 | 0.371 |
|  | Livestock ownership | 0.247 |  | 0.278 |  | 0.287 |  |
|  | %access communication device | 0.432 |  | 0.45 |  | 0.568 |  |
|  | Saving and loan associations | 0.07 |  | 0.04 |  | 0.09 |  |
| Livelihood strategy | Diversity of income sources | 0.315 | 0.343 | 0.44 | 0.444 | 0.325 | 0.385 |
|  | Information exchange/SSS/ | 0.46 |  | 0.495 |  | 0.553 |  |
|  | Coping strategies | 0.179 |  | 0.36 |  | 0.484 |  |
|  | Technology utilization | 0.42 |  | 0.48 |  | 0.18 |  |
| Social capital | Conflict management | 0.558 | 0.505 | 0.551 | 0.499 | 0.621 | 0.542 |
|  | vertical linkages (involvement in decision) | 0.453 |  | 0.449 |  | 0.463 |  |
| Access to basic services | market | 0.38 | 0.35 | 0.36 | 0.327 | 0.44 | 0.355 |
|  | health services | 0.4 |  | 0.48 |  | 0.32 |  |
|  | primary school | 0.66 |  | 0.54 |  | 0.52 |  |
|  | all weather road | 0.18 |  | 0.051 |  | 0.18 |  |
|  | saving and credit | 0.43 |  | 0.48 |  | 0.42 |  |
|  | electricity | 0.05 |  | 0.051 |  | 0.25 |  |
