# Supplementary material for: Exploring households’ resilience to climate change-induced shocks using Climate Resilience Index in Dinki watershed, central highlands of Ethiopia
Source: PLoS One. 2019 Jul 9;14(7):e0219393. doi: 10.1371/journal.pone.0219393 (PMC6615616; doi:10.1371/journal.pone.0219393)
Supplement: S2 Table — (DOCX) [file pone.0219393.s002.docx]

S 2 Table. Bivariate correlation of variables in the highland agro-ecology (RI=resilience index, CC= climate change, IncomeD=income diversity, SSS=social support score, SWC=soil and water conservation, EWS=early warning system, wSuff= water sufficiency, wConflict= water conflict).

|  | RI | Family size | topography | Injury | CC imp | Farm | IncomeD. | livestock | SSS | coping | SWC | EWS | Market | Road | credit | Device | sharing | wSuff. | wConflict |
| --- | --- | --- | --- | --- | --- | --- | --- | --- | --- | --- | --- | --- | --- | --- | --- | --- | --- | --- | --- |
| RI |  | -0.18 | 0.06 | 0.59** | -0.55** | 0.69** | 0.68** | 0.89** | 0.72** | 0.75** | 0.65** | o.45** | 0.68** | 0.04 | 0.64** | 0.84** | 0.82** | 0.68** | 0.39** |
| Family size |  |  | -0.07 | -0.16 | 0.22* | -0.15 | -0.011 | -0.04 | -0.26* | -0.19 | -0.09 | -0.05 | -0.05 | -0.03 | -0.10 | -0.03 | -0.09 | -0.20* | -0.21* |
| topography |  |  |  | 0.10 | -0.14 | 0.12 | 0.11 | 0.09 | 0.17 | 0.13 | 0.12 | 0.12 | 0.09 | -0.2* | 0.12 | 0.10 | 0.11 | 0.12 | 0.08 |
| Injury/death |  |  |  |  | -0.32** | 0.29** | 0.41** | 0.63** | 0.40** | 0.61** | 0.42** | 0.17 | 0.73** | -.006 | 0.51** | 0.49** | 0.43** | 0.61** | 0.29** |
| CC impacts |  |  |  |  |  | -0.41** | -0.50** | -0.36** | -0.49** | -0.19 | -0.46** | -0.15 | -0.24* | -0.08 | -0.19 | -0.29** | -0.32** | -0.30** | -0.16 |
| Farm size |  |  |  |  |  |  | 0.38** | 0.54** | 0.47** | 0.45** | 0.34** | 0.33** | 0.49** | -0.04 | 0.44** | 0.6** | 0.57** | 0.54** | 0.36** |
| Income diversity |  |  |  |  |  |  |  | 0.45** | 0.45** | 0.37** | 0.9** | 0.23** | 0.41** | -0.13 | 0.29** | 0.4** | 0.41** | 0.37** | 0.29** |
| livestock |  |  |  |  |  |  |  |  | 0.62** | 0.79** | 0.43** | 0.35** | 0.63** | 0.002 | 0.67** | 0.74** | 0.73** | 0.61** | 0.25* |
| SSS |  |  |  |  |  |  |  |  |  | 0.58** | 0.39** | 0.29** | 0.4** | -0.04 | 0.42** | 0.54** | 0.61** | 0.50** | 0.34** |
| Coping strategies |  |  |  |  |  |  |  |  |  |  | 0.37** | 0.33** | 0.56** | -0.08 | 0.64** | 0.67** | 0.64** | 0.54** | 0.28** |
| SWC |  |  |  |  |  |  |  |  |  |  |  | 0.27** | 0.41** | -0.07 | 0.25* | 0.39** | 0.37** | 0.38** | 0.26** |
| EWS |  |  |  |  |  |  |  |  |  |  |  |  | .28** | -0.4 | 0.28** | 0.43** | 0.39** | 0.22** | 0.04 |
| Market |  |  |  |  |  |  |  |  |  |  |  |  |  | -0.11 | 0.50** | 0.59** | 0.49** | 0.65** | 0.28** |
| Road |  |  |  |  |  |  |  |  |  |  |  |  |  |  | 0.07 | 0.02 | 0.07 | -0.03 | -0.13 |
| Credit |  |  |  |  |  |  |  |  |  |  |  |  |  |  |  | 0.59** | 0.56** | 0.47** | 0.22* |
| Device |  |  |  |  |  |  |  |  |  |  |  |  |  |  |  |  | 0.89** | 0.53** | 0.23* |
| sharing |  |  |  |  |  |  |  |  |  |  |  |  |  |  |  |  |  | 0.49** | 0.20* |
| Water s-ufficiency |  |  |  |  |  |  |  |  |  |  |  |  |  |  |  |  |  |  | 0.51** |
| Water conflict |  |  |  |  |  |  |  |  |  |  |  |  |  |  |  |  |  |  |  |

**significant at 0.01 level; *significant at 0.05 level
