# Supplementary material for: Exploring households’ resilience to climate change-induced shocks using Climate Resilience Index in Dinki watershed, central highlands of Ethiopia
Source: PLoS One. 2019 Jul 9;14(7):e0219393. doi: 10.1371/journal.pone.0219393 (PMC6615616; doi:10.1371/journal.pone.0219393)
Supplement: S3 Table — (DOCX) [file pone.0219393.s003.docx]

S 3 Table. Bivariate correlation of variables in the midland agro-ecology (partGonce= participation in governance)

|  | RI | Infertile soil | shock | Farm size | Income divers | livestock | Com device | EWS | partGonce | wsuffi | wconflict | Health | Road | school | CC impact | Gender |
| --- | --- | --- | --- | --- | --- | --- | --- | --- | --- | --- | --- | --- | --- | --- | --- | --- |
| RI |  | 0.31** | .046** | 0.62** | 0.39** | 0.69** | 0.72** | 0.28** | 0.30** | 0.62** | 0.42** | 0.49** | 0.06 | 0.12 | 0.58** | 0.49** |
| Infertile soil |  |  | 0.21* | 0.14 | 0.79** | 0.39** | 0.29** | 0.20* | 0.25* | 0.13 | -0.001 | 0.25* | -0.01 | 0.09 | 0.19 | 0.21* |
| Shock events |  |  |  | 0.42** | 0.16 | 0.52** | 0.48** | 0.19 | 0.08 | 0.35** | 0.20* | 0.44** | -0.11 | 0.14 | 0.19 | 0.08 |
| Farm size |  |  |  |  | 0.12 | 0.48** | 0.45** | 0.22* | 0.08 | 0.29** | 0.12 | 0.29** | -0.07 | -0.04 | 0.54** | 0.36** |
| Income diversity |  |  |  |  |  | 0.43** | 0.25* | 0.06 | 0.30** | 0.16 | 0.03 | 0.19 | 0.11 | -0.02 | 0.25* | 0.33** |
| livestock |  |  |  |  |  |  | 0.58** | 0.16 | 0.14 | 0.38** | 0.23* | 0.35** | 0.09 | 0.02 | 0.25* | 0.27** |
| Device access |  |  |  |  |  |  |  | 0.56** | 0.12 | 0.42** | 0.16 | .036** | -0.04 | 0.01 | 0.25* | 0.27** |
| EWS |  |  |  |  |  |  |  |  | 0.04 | 0.03 | -0.04 | 0.09 | -0.16 | -0.18 | 0..03 | 0.04 |
| Participation in governance |  |  |  |  |  |  |  |  |  | 0.04 | 0.13 | 0.28** | 0.17 | -0.09 | 0.13 | 0.13 |
| Water sufficiency |  |  |  |  |  |  |  |  |  |  | 0.57** | 0.27** | -0.06 | 0.14 | 0.21* | 0.17 |
| Water conflict |  |  |  |  |  |  |  |  |  |  |  | 0.25* | -0.001 | -0.01 | 0.09 | 0.04 |
| Health access |  |  |  |  |  |  |  |  |  |  |  |  | -0.17 | 0.20* | 0.23* | 0.15 |
| Road access |  |  |  |  |  |  |  |  |  |  |  |  |  | -0.05 | -0.08 | -0.03 |
| School access |  |  |  |  |  |  |  |  |  |  |  |  |  |  | 0.03 | 0.04 |
| CC impacts |  |  |  |  |  |  |  |  |  |  |  |  |  |  |  | 0.56** |
| gender |  |  |  |  |  |  |  |  |  |  |  |  |  |  |  |  |

**significant at 0.01 level

*significant at 0.05 level
