# Supplementary material for: Exploring households’ resilience to climate change-induced shocks using Climate Resilience Index in Dinki watershed, central highlands of Ethiopia
Source: PLoS One. 2019 Jul 9;14(7):e0219393. doi: 10.1371/journal.pone.0219393 (PMC6615616; doi:10.1371/journal.pone.0219393)
Supplement: S4 Table — (DOCX) [file pone.0219393.s004.docx]

S 4 Table. Bivariate correlation of variables in the lowland agro-ecology (CBOs=community-based organizations).

|  | RI | Injury/death | Coping strategies | Farm size | livestock | Growing of perennial crops | CBOs | gender | market |
| --- | --- | --- | --- | --- | --- | --- | --- | --- | --- |
| RI |  | -0.22* | 0.77** | 0.65** | 0.70** | 0.20* | 0.71** | 0.42** | 0.34** |
| Injury/death events |  |  | 0.01 | 0.05 | 0.05 | 0.07 | -0.03 | -0.06 | -0.13 |
| Coping strategies |  |  |  | 0.59** | 0.73** | 0.20 | 0.69** | 0.23* | 0.19 |
| Farm size |  |  |  |  | 0.60** | 0.16 | 0.56** | 0.38** | 0.02 |
| livestock |  |  |  |  |  | 0.06 | 0.61** | 0.20 | 0.13 |
| Perennial crops |  |  |  |  |  |  | 0.14 | 0.16 | -0.21* |
| Membership to CBOs |  |  |  |  |  |  |  | 0.33** | 0.19 |
| Gender |  |  |  |  |  |  |  |  | 0.09 |
| Market access |  |  |  |  |  |  |  |  |  |

**significant at 0.01 level

*significant at 0.05 level
