# Supplementary material for: Exploring households’ resilience to climate change-induced shocks using Climate Resilience Index in Dinki watershed, central highlands of Ethiopia
Source: PLoS One. 2019 Jul 9;14(7):e0219393. doi: 10.1371/journal.pone.0219393 (PMC6615616; doi:10.1371/journal.pone.0219393)
Supplement: S5 Table — (DOCX) [file pone.0219393.s005.docx]

S 5 Table. Bivariate correlation of variables in the combined data

|  | RI | Family size | shock | sharing | Gender | livestock | Farm size | CBO | SSS | SWC | Income  Div. | Device | Market | Road | credit | Coping | EWS | Age |
| --- | --- | --- | --- | --- | --- | --- | --- | --- | --- | --- | --- | --- | --- | --- | --- | --- | --- | --- |
| RI |  | -0.32** | 0.24** | 0.44** | 0.44** | 0.77** | 0.68** | 0.64** | 0.68** | 0.57** | 0.56** | 0.56** | 0.51** | 0.14* | 0.52** | 0.74** | 0.29** | 0.45** |
| Family size |  |  | -0.11 | -0.04 | -0.11 | -0.14* | -0.31** | -0.27** | -0.30** | -0.09 | -0.06 | -0.01 | -0.15* | -0.01 | -0.11 | -0.28** | 0.02 | -0.10 |
| Shock events |  |  |  | -0.09 | 0.03 | 0.38** | 0.23** | 0.42** | 0.34** | 0.21** | 0.23** | 0.26** | 0.17** | -0.16** | 0.17** | 0.35** | 0.09 | 0.14* |
| sharing |  |  |  |  | 0.12* | 0.33** | 0.23** | 0.25** | 0.26** | 0.09 | 0.15* | 0.37** | 0.27** | 0.12* | 0.22** | 0.24** | 0.21** | 0.08 |
| Gender |  |  |  |  |  | 0.24** | 0.34** | 0.27** | 0.35** | 0.28** | 0.29** | 0.18** | 0.12* | 0.02 | 0,11 | 0.17** | 0.07 | 0.41** |
| livestock |  |  |  |  |  |  | 0.54** | 0.57** | 0.59** | 0.37** | 0.40** | 0.42** | 0.37** | 0.03 | 0.44** | 0.74** | 0.17** | 0.26** |
| farm size |  |  |  |  |  |  |  | 0.46** | 0.49** | 0.28** | 0.29** | 0.34** | 0.28** | -0.07 | 0.34** | 0.51** | 0.19** | 0.40** |
| CBO |  |  |  |  |  |  |  |  | 0.59** | 0.24** | 0.29** | 0.37** | 0.39** | 0.03 | 0.35** | 0.63** | 0.15** | 0.33** |
| SSS |  |  |  |  |  |  |  |  |  | 0.32** | 0.34** | 0.32** | 0.36** | -0.03 | 0.40** | 0.58** | 0.12* | 0.52** |
| SWC |  |  |  |  |  |  |  |  |  |  | 0.85** | 0.26** | 0.18** | -0.05 | 0.22** | 0.32** | 0.19** | 0.31** |
| Income Diversity |  |  |  |  |  |  |  |  |  |  |  | 0.26** | 0.19** | -0.09 | 0.25** | 0.34** | .14* | 0.30** |
| Device |  |  |  |  |  |  |  |  |  |  |  |  | 0.37** | 0.08 | 0.36** | 0.43** | 0.37** | 0.14* |
| Market |  |  |  |  |  |  |  |  |  |  |  |  |  | 0.13* | 0.36** | 0.40** | 0.04 | 0.14* |
| Road |  |  |  |  |  |  |  |  |  |  |  |  |  |  | -0.09 | 0.05 | -0.04 | -0.07 |
| Credit |  |  |  |  |  |  |  |  |  |  |  |  |  |  |  | 0.48** | 0.17** | 0.17** |
| coping |  |  |  |  |  |  |  |  |  |  |  |  |  |  |  |  | 0.17** | 0.20** |
| EWS |  |  |  |  |  |  |  |  |  |  |  |  |  |  |  |  |  | -0.01 |
| Age |  |  |  |  |  |  |  |  |  |  |  |  |  |  |  |  |  |  |

**significant at 0.01 level

*significant at 0.05 level
